# Locked nucleic acid-modified antisense oligonucleotides attenuate scar hyperplasia through targeted inhibition of CTGF

**DOI:** 10.3389/fphar.2025.1623640

**Published:** 2025-08-21

**Authors:** Jinhe Li, Xi Wu, Ying Yang, Ruiqi Mao, Zherui Li, Xiujun Zhang, Wenguo Wei, Wendi Wang, Hailong Li, Honggang Zhou, Cheng Yang

**Affiliations:** ^1^ State Key Laboratory of Medicinal Chemical Biology, College of Pharmacy, Nankai University, Tianjin, China; ^2^ Department of Dermatology, Tianjin Academy of Traditional Chinese Medicine Affiliated Hospital, Tianjin, China; ^3^ Department of Dermatology, Tianjin First Central Hospital, School of Medicine, Nankai University, Tianjin, China; ^4^ Department of Plastic and Burn Surgery, Tianjin First Central Hospital, Tianjin, China; ^5^ Nankai International Advanced Research Institute (Shenzhen Futian), ShenZhen, China

**Keywords:** CTGF, antisense oligonucleotide, scar, LNA, fibrosis

## Abstract

Connective tissue growth factor (CTGF) is notably upregulated in scar tissue, making it a promising target for therapeutic intervention. Here, we have designed and screened an antisense oligonucleotide (ASO) that binds specifically to the exon five sequence of CTGF, with particular emphasis on the use of 2′-O-methoxyethyl (MOE) and locked nucleic acid (LNA) modifications to enhance stability and specificity. *In vitro* experiments demonstrated that both MOE-ASO#1 and LNA-ASO#1 significantly inhibited fibroblast proliferation and extracellular matrix protein expression. *In vivo* studies using mouse and rabbit scar models, as well as a nude mouse keloid xenograft model, revealed that these ASOs effectively reduced scar formation and keloid growth while also suppressing IL-6 expression. LNA-ASO#1 showed superior pharmacodynamics compared to MOE-ASO#1. Mechanistic investigations indicated that the ASOs exert their antifibrotic effects by inhibiting the TGF-β1 pathway, myofibroblast activation, and extracellular matrix production. These findings suggest that LNA-ASO#1 is a promising therapeutic strategy for the treatment of scars.

## 1 Introduction

Scar formation represents a complex physiological response to skin injury (Jiang et al., 2007; Gauglitz et al., 2011; Ogawa, 2018). Following tissue damage, inflammatory cells, including macrophages and lymphocytes, infiltrate the wound site and release cytokines and growth factors that trigger fibroblast activation—a key driver of scar development. While inflammation is essential for initial wound healing, excessive fibroblast proliferation and dysregulated extracellular matrix (ECM) deposition, particularly the overproduction of type I and III collagen, are central to pathological scarring. This imbalance can lead to the formation of hypertrophic scars or keloids. Pathological scars not only cause physical discomfort—such as itching, pain, and erythema—but also significantly impair aesthetics and function, severely impacting patients’ quality of life (Ogawa, 2017; Ekstein et al., 2021; Zheng et al., 2023). Therefore, developing effective scar treatments is a critical area of medical research.

The TGF-β1 signaling pathway plays a central role in fibrosis and scar formation, and its key role has been widely confirmed (Kim et al., 2018). However, despite its importance, the progress of candidate drugs directly targeting the TGF-β1 pathway in early clinical trials has been limited, prompting researchers to explore alternative strategies. CTGF (connective tissue growth factor) is a cysteine-rich polypeptide and a key effector of the TGF-β1 signaling pathway (Jiang et al., 2007). By interacting with TGF-β1 receptors, it enhances the fibrotic effects of TGF-β1, promoting extracellular matrix deposition and thereby exacerbating scar formation.

The CTGF gene is located on chromosome 6q23.1 and consists of five exons (Fu et al., 2022). Exon 1 encodes a signal peptide, while exons two to five encode four distinct structural domains. Studies have shown that the CT domain encoded by exon five plays a crucial role in regulating CTGF’s functions, including cell adhesion, migration, proliferation, and the synthesis and deposition of ECM, making it a key regulatory region in the fibrotic process (Perbal, 2004; Amano et al., 2023). Notably, Hiroaki Amano and colleagues found that CTGF knock-in mice with exon five deletion developed normally and did not experience premature death due to widespread tissue inflammation, as seen in TGF-β1 knockout mice (Amano et al., 2023; Jin et al., 2024). This finding suggests that CTGF may be a more selective intervention target, capable of specifically regulating connective tissue formation in wound healing or fibrotic diseases, while avoiding the broad side effects that could arise from directly targeting TGF-β. Therefore, targeting CTGF, particularly its CT domain, provides a new direction for research into the treatment of fibrotic diseases.

With advancements in antisense oligonucleotide (ASO) technology, ASO therapy has gained recognition (Bennett, 2019; Kim, 2023). Antisense therapy has been used clinically f (Amano et al., 2023) or conditions such as cytomegalovirus retinitis, Duchenne muscular dystrophy (Finkel et al., 2017; Randeree and Eslick, 2018; Migliorati et al., 2022). In this context, ASOs targeting specific genes have become an attractive new therapeutic strategy (Burbano et al., 2022; Ma et al., 2022). ASOs are short, synthetic single-stranded DNA/RNA-like molecules designed to selectively bind RNA and regulate protein expression. Unlike drugs that bind directly to proteins, ASOs modulate target RNA processing, translation, or degradation, allowing precise control over target mRNA and protein expression without the limitations of protein druggability. Therefore, developing ASOs targeting CTGF holds promise for breakthrough scar treatments.

In this study, we exploited the specificity of ASO technology to target and inhibit the expression of CTGF to reduce scarring. To enhance the stability and nuclease resistance of ASO, we adopted a Gamper design approach that incorporated modifications such as 2′-O-methoxyethyl (MOE) and locking nucleic acid (LNA). The therapeutic efficacy of these modified ASOs was evaluated through a series of *in vitro* and *in vivo* experiments that evaluated their effects on fibroblast proliferation, ECM protein expression, and key signaling pathways involved in fibrosis. Our results provide strong preclinical evidence in favor of LNA-ASO#1 as a promising scar treatment strategy. These findings provide the basis for further clinical research and optimization of ASO-based therapies for fibrotic diseases.

## 2 Materials and methods

### 2.1 Design and synthesis of antisense oligonucleotides

The CTGF exon five sequence was retrieved from the NCBI database, and ASOs targeting this sequence were designed. The gamper design method was used, incorporating modifications such as MOE, LNA, and PS backbone to enhance the stability, affinity, and specificity of the ASOs. All ASO sequences were synthesized by Tsingke Biotech Co., Ltd., with the specific sequences listed in Table 1. The ASOs were dissolved in sterile PBS without Ca^2+^ and Mg^2+^, centrifuged (10,000 rpm at room temperature for 1–2 min), and filtered through a 0.22 μm filter.

**TABLE 1:** Oligonucleotide sequences for scar research.

ASO name	Length	Position	Sequence 5′-3′
ASO#1	21	1,016–1,037	TAT​GTC​TTC​ATG​CTG​GTG​CAG
ASO#2	21	1,127–1,148	TTC​TTC​TTC​ATG​ACC​TCG​CCG
ASO#3	19	1,144–1,163	TTG​ATG​AAC​ATC​ATG​TTC​T
ASO#4	21	1,188–1,209	AAA​GAT​GTC​ATT​GTC​TCC​GGG
ASO#5	21	1,253–1,274	TTA​ATG​TCT​CTC​ACT​CTC​TGG
ASO#6	21	1,433–1,454	TAA​CAT​TCT​TCA​AAC​CAG​TGT
ASO#7	21	1,686–1707	TTA​AGG​AAC​AAC​TTG​ACT​CAG
ASO#8	19	1743–1762	TTC​CTG​AAC​AGT​GTC​ATT​C
ASO#9	21	1888–1909	TAA​ATT​AAC​TTA​GAT​AAC​TGT
ASO#10	21	1976–1997	TTA​CAT​TCT​ACC​TAT​GGT​GTT
ASO#11	21	1995–2016	TTG​AAC​GAT​CAG​ACA​AGC​TTT
Scrambled -ASO	21		TTC​GGT​GAT​CTA​GCT​GTG​ACT

### 2.2 Animal models

#### 2.2.1 Mouse hypertrophic scar model

Male C57BL/6 mice were divided into four groups: control, model, MOE-ASO#1 treatment, and LNA-ASO#1 treatment (5 mice per group). The control group received no treatment. An 8 mm circular wound was made on the backs of mice in the model and treatment groups, and a 10 mm silicone ring was sutured onto the wound to apply tension. After 2 weeks, when scars had formed, a 10 μL intralesional injection was administered using a 31G syringe. The model group received intralesional saline, while the treatment groups received MOE-ASO#1 or LNA-ASO#1 (0.5 mg/kg), twice weekly for 4 weeks. Mice were then euthanized, and scar tissues were collected for analysis.

#### 2.2.2 Rabbit ear hypertrophic scar model

Female New Zealand rabbits were divided into four groups: control, model, MOE-ASO#1 treatment, and LNA-ASO#1 treatment (2 rabbits per group). The control group received no treatment. In the model and treatment groups, six 8 mm circular wounds were made on the ventral side of each rabbit’s ear using an 8 mm punch biopsy. After 4 weeks, when scars had formed, a 10 μL intralesional injection was administered using a 31G syringe. The model group received intralesional saline, while the treatment groups received MOE-ASO#1 or LNA-ASO#1 (10 µg/scar), twice weekly for 4 weeks. Rabbits were then euthanized for data collection.

#### 2.2.3 Nude mouse keloid xenograft model

Male BALB/c nude mice were divided into three groups: model, MOE-ASO#1 treatment, and LNA-ASO#1 treatment (3 mice per group). Fresh keloid tissue fragments (5 × 5 × 5 mm, weighing 0.07–0.1 g) were implanted into incisions on both sides of the axilla. Post-surgery, the wounds were secured with pressure dressings. Fourteen days post-implantation, injection was performed using a 10 μL volume administered intralesionally with a 31G syringe. The treatment groups received MOE-ASO#1 or LNA-ASO#1 (0.5 mg/kg) twice weekly for 4 weeks, while the model group received saline injections. Mice were euthanized after the treatment period, and keloid tissues were collected for analysis.

### 2.3 Human sample

Human skin tissues from scar patients and normal controls were collected from Department of Plastic and Burn Surgery, Tianjin First Central Hospital. Keloid patients were diagnosed based on standard criteria. The clinical characteristics of the subjects are shown in Table 2.

**TABLE 2:** Clinical characteristics of the study group.

Patient gender	Age	Diagnosed disease	Scar location
Female	66	Keloid	Right auricle
Male	60	Keloid	Behind left ear
Female	34	Keloid	Right auricle
Female	67	Hypertrophic scar	Right shoulder
Female	45	Hypertrophic scar	Right zygomatic area

### 2.4 Immunofluorescence

Paraffin-embedded tissue sections were deparaffinized and subjected to antigen retrieval with sodium citrate solution (Solarbio). Sections were blocked and incubated overnight at 4 °C with primary antibody (CTGF, Cell Signaling Technology), followed by incubation with secondary antibodies (Affinity) at room temperature for 1 h. Sections were mounted with DAPI-containing anti-fade medium (YEASEN) and imaged using a confocal microscope (ZEISS LSM800).

### 2.5 Cell viability assay (CCK-8)

Cells were seeded into 96-well plates, and after 24 h of incubation, cells were pretreated with various concentrations (0,10,50,100,250,500,1000 nM) of ASO#1, Scrambled-ASO, MOE-ASO#1, LNA-ASO#1. Then, 10 µL of CCK-8 solution (YEASEN) was added to each well, and the cells were incubated for an additional 2 h. The absorbance (OD value) at 450 nm was measured using a microplate reader.

### 2.6 Hydroxyproline content detection

Skin tissues were homogenized in saline to create a 10% homogenate. Hydroxyproline content was measured using a hydroxyproline assay kit (Solarbio) according to the manufacturer’s instructions.

### 2.7 EdU cell proliferation assay

After 24 h of cell treatment, EdU reagent (Beyotime) was added and incubated for 2 h. Cells were washed, fixed, and incubated with Click reaction solution for 30 min. EdU detection and Hoechst 33,342 staining were performed, and images were captured using a fluorescence microscope (ZEISS LSM800).

### 2.8 Primary mouse skin fibroblast extraction

Skin samples were collected from euthanized mice and digested with trypsin (Solarbio) overnight at 4 °C. Dermal layers were then incubated with collagenase (Solarbio) at 37 °C, followed by DNase treatment. The samples were centrifuged to isolate fibroblasts.

### 2.9 ASO transfection

At 70% confluence, cells were transfected with ASOs using Lipo8000(Beyotime) according to the kit instructions. The cells were then incubated for 48 h before proceeding with subsequent experiments.

### 2.10 FAM-labeled ASO fluorescence assay

All FAM-labeled ASOs were synthesized by Tsingke Biotech Co., Ltd. FAM-labeled ASOs were transfected into cells using Lipo8000 (Beyotime) and incubated for 1, 3, 5, and 7 days. At the end of each incubation period, cells were fixed with 4% paraformaldehyde. Cells were stained with DAPI(YEASEN) followed by three washes with PBS, and FAM fluorescence was observed using a confocal microscope (ZEISS LSM800).

### 2.11 Scar elevation index (SEI)

Rabbit ear scar samples were cut from the most elevated point, and the vertical distance from the highest point of the scar to the cartilage surface and from the ventral skin surface to the cartilage surface was measured. SEI was calculated as SEI = (vertical distance from the highest point of the scar to the cartilage surface)/(vertical distance from the ventral skin surface to the cartilage surface).

### 2.12 Real-time quantitative PCR

Total RNA was extracted from cell or skin tissue samples using TRIzol (Solarbio). cDNA was synthesized using a reverse transcription kit. Quantitative PCR was performed using SYBR Green premix (YEASEN), and analysis was conducted on a real-time PCR system. The primer sequences used in qRT-PCR is shown in Table 3. PCR conditions were: 95 °C for 15 min, followed by 40 cycles of 95 °C for 10 s, 58 °C for 30 s, and 72 °C for 30 s. GAPDH was used as the reference gene, and relative gene expression was calculated using the 2^−ΔΔCT^ method.

**TABLE 3:** The primer sequences used in qRT-PCR.

Gene name	Primer (forward/reverse)	Sequence (5′→3′)
α-SMA α-SMA	Forward	GTC​GAA​TGC​AAC​AAG​GAA​GCC
	Reverse	TGG​GTG​AAC​TCC​ATC​GCT​GTA
Col-1α	Forward	AGG​GCA​GGG​AAC​AAC​TTG​ATG
Col-1α	Reverse	GGA​CTG​ACC​AAG​ATG​GGA​ACA
GAPGH	Forward	GCG​CCC​AAT​ACG​ACC​AAA​TC
GAPGH	Reverse	GAC​AGT​CAG​CCG​CAT​CTT​CT
Fn	Forward	AGA​CCA​GCT​CAG​GGT​GTT​G
Fn	Reverse	GCA​TCA​ACT​TGG​AAG​CCA​GT
CTGF	Forward	ACA​CAA​CAA​CTC​TTC​CCC​GC
CTGF	Reverse	TGCAGTTCTGGCCGACG
Col-3α	Forward	GGT​GAG​CCT​GGT​CAA​ACG​G
Col-3α	Reverse	ACT​GTG​TCC​TTT​CAC​GCC​TTT

### 2.13 Western blot

Cell or skin tissue samples were lysed in RIPA buffer (Beyotime) and centrifuged to extract total protein. Protein samples were separated by SDS-PAGE, transferred to PVDF membranes (Millipore), blocked, and incubated with primary antibodies overnight at 4 °C, followed by incubation with secondary antibodies at room temperature. Protein detection was performed using an ECL kit (YEASEN) and visualized. For Western blotting analysis, the following primary antibodies were used: CTGF, Col-1α, Col-3α,α-SMA, Fn and β-tubulin (Cell Signaling Technology); TGF-β1,p-Smad2/3, Smad2/3, p-AKT, AKT, p-ERK, ERK, p-PI3K and PI3K (Affinity).

### 2.14 Wound healing assay

Draw three parallel horizontal lines in the center of a 12-well plate. Once KF cells reach confluency, create vertical scratches with a yellow pipette tip. Wash dislodged cells with PBS and incubate in drug-containing medium. Collect samples at 24 h and capture images at three fixed positions for each group. Analyze using ImageJ. Healing Rate = (Initial Scratch Area - Scratch Area at Time t)/Initial Scratch Area × 100%.

### 2.15 Statistical analysis

Data were analyzed using GraphPad Prism 9.0 and are presented as mean ± SD. The normality of data distribution within each group was assessed using the Shapiro-Wilk test. Comparisons between two groups were performed using the independent samples t-test, and comparisons among multiple groups were made using one-way ANOVA, followed by Tukey’s post-hoc test for pairwise comparisons. p < 0.05 was considered statistically significant.

## 3 Results

### 3.1 Identification of potent CTGF-targeting ASO candidates through systematic screening

**FIGURE 1:**
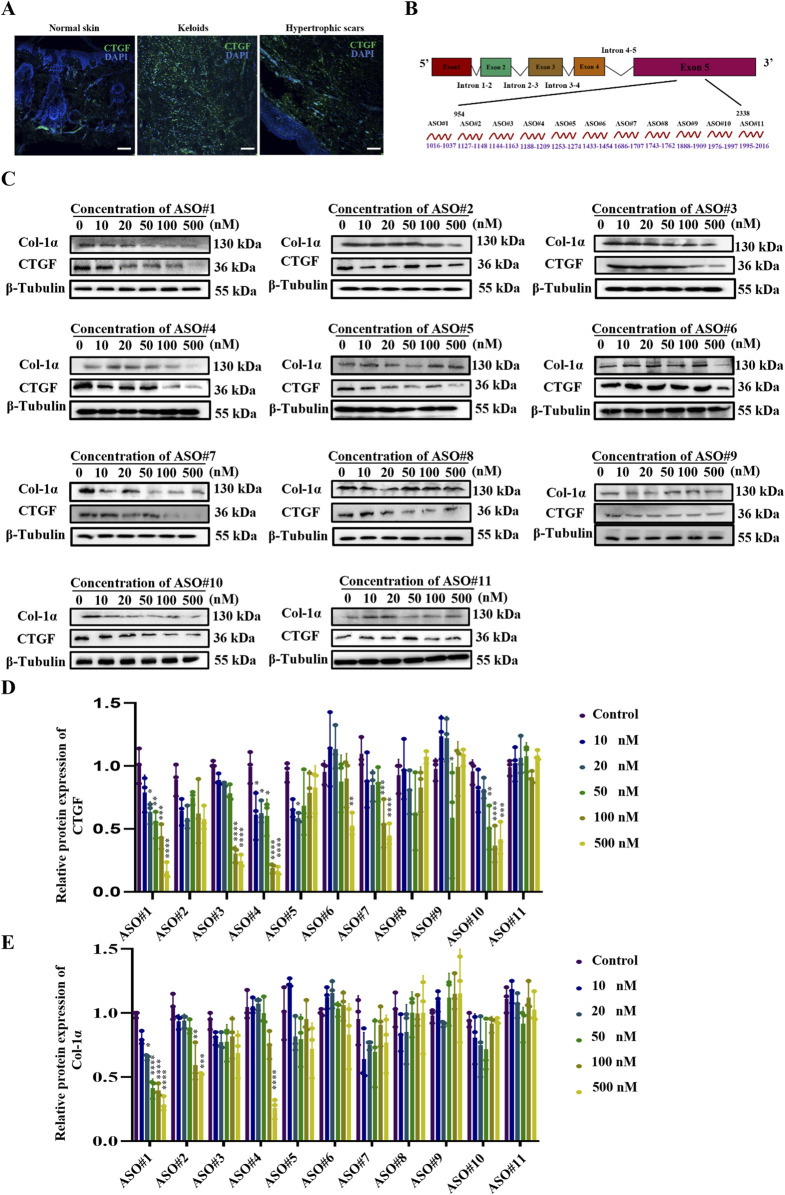
Identification of Potent CTGF-Targeting ASO Candidates Through Systematic Screening **(A)** CTGF expression in human healthy skin as well as hypertrophic scars and keloids. Scale bar = 25 μm. **(B)** Schematic diagram of CTGF antisense oligonucleotide design. **(C)** Protein expression of CTGF and Col-1α under the concentration gradient of the CTGF-ASO series. **(D)** Statistical results of CTGF protein expression. **(E)** Statistical results of Col-1α protein expression. p < 0.05, **p < 0.01, ***p < 0.001, ****p < 0.0001.

Immunofluorescence analysis (Figure 1A) using human pathological scars confirmed marked CTGF overexpression, supporting its therapeutic potential (Khoo et al., 2006; Qiu et al., 2024). Based on this finding, we rationally designed 11 antisense oligonucleotides (ASOs) targeting exon five of CTGF for systematic evaluation (Figure 1B).

Given that fibroblasts are key effector cells in scar pathology (Dong et al., 2022; Talbott et al., 2022), we isolated primary fibroblasts from mouse skin (PSF) for preliminary ASO screening (Li et al., 2021). The experimental results showed that upon transfecting these fibroblasts with ASOs, the scrambled ASO treatment group did not exhibit a significant reduction in CTGF protein expression (Supplementary Figures S1A-C). In contrast, ASO#1 demonstrated excellent inhibitory effects, achieving a half-maximal inhibitory concentration (IC50) of 50 nM with a strong dose-dependent response (Figures 1C,D). To further evaluate the time-dependent effects, we measured the impact of 50 nM ASO#1 on CTGF protein expression levels at different time points (12, 24, 48, 72, and 96 h). The results (Supplementary Figure S2) showed that CTGF protein expression was significantly reduced 48 h after transfection. This indicates that ASO#1 achieved significant inhibition at the 48-h time point, providing a critical reference for subsequent studies.

During scar formation, the synthesis and deposition of Col-1α increase significantly, influencing scar tissue development (Sharma et al., 2022; Xu et al., 2022). To further assess the inhibitory effect of ASOs on fibroblast activation, we measured protein levels of Col-1α. Notably, consistent with CTGF expression, CTGF-ASO#1 exhibited dose-dependent inhibition of type I collagen (Figures 1C,E).

### 3.2 Enhanced stability and efficacy of MOE/LNA-ASO#1 in inhibiting PSF cell proliferation and ECM protein expression

**FIGURE 2:**
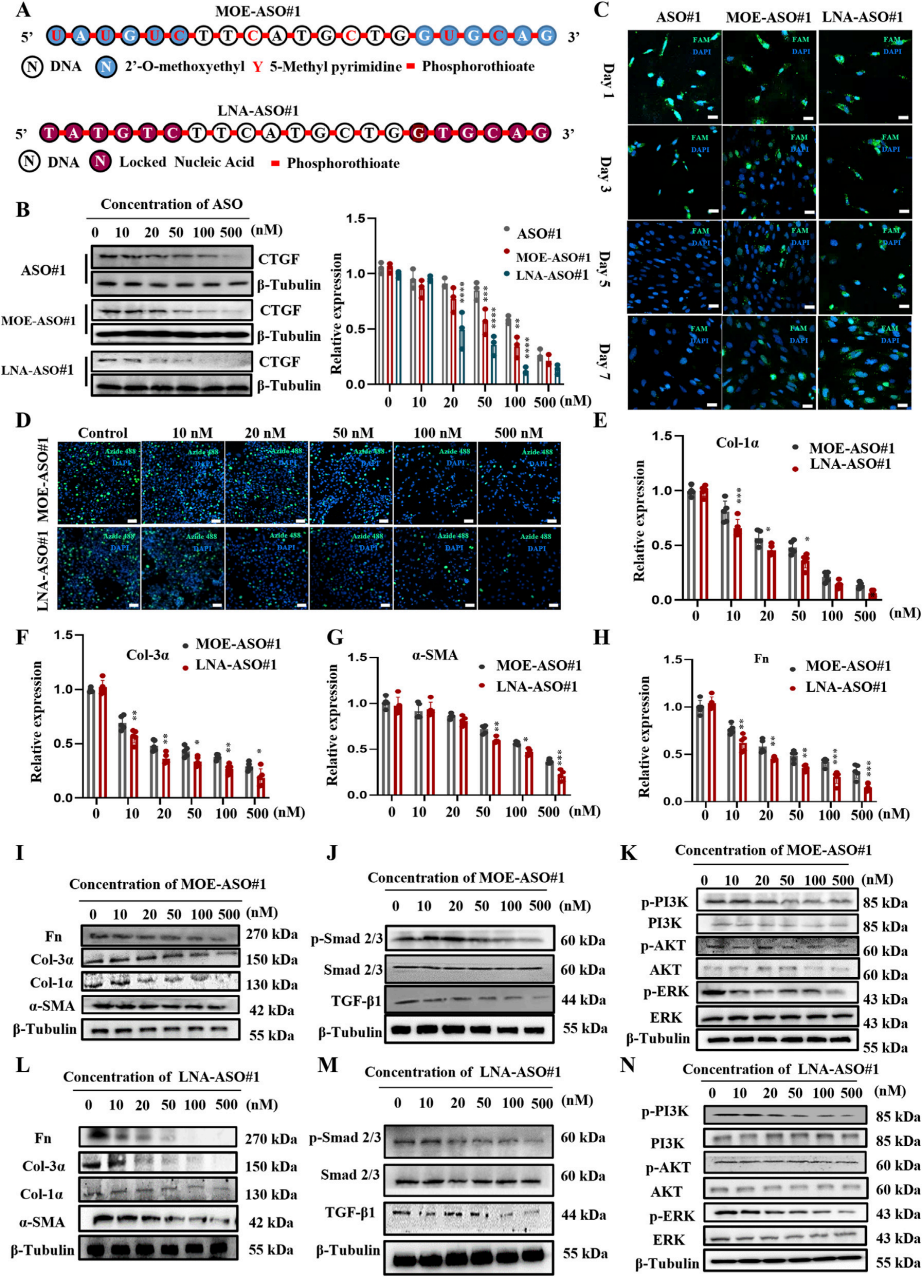
Enhanced Stability and Efficacy of MOE/LNA-ASO#1 in Inhibiting PSF Cell Proliferation and ECM Protein Expression **(A)** Schematic diagram of MOE and LNA modification. Effects of MOE, LNA and unmodified ASO#1 on CTGF protein **(B)** expression in PSF cells and comparison of fluorescence intensity **(C)**. **(D)** EdU experiment to detect the effect of concentration gradient ASO on cell proliferation. Effects of ASO concentration gradient on the expression of Col-1α **(E)**, Col-3α **(F)**, α -SMA **(G)**, FN **(H)** mRNA and protein **(I-L)** Effects of concentration gradient ASO on Smad 2/3 **(J-M)** PI3K, AKT and ERK phosphorylation levels **(K-N)** signaling pathway proteins. Scale bar = 25 μm*p < 0.05, **p < 0.01, ***p < 0.001, ****p < 0.0001.

Unmodified nucleic acid drugs have significant drawbacks, including instability *in vivo*, rapid degradation by nucleases upon entering the bloodstream, and quick clearance by the kidneys, resulting in a short half-life (Chan et al., 2006; Ramasamy et al., 2022). The Gamper design method optimizes ASOs for better targeting efficiency and specificity. As shown in Figure 2A, we modified the initial sequences with MOE and LNA (Bockstahler et al., 2022; Zhu et al., 2022). We transfected the modified CTGF-ASOs into PSF to observe their impact on CTGF protein expression. Compared to unmodified sequences, MOE and LNA modifications significantly improved the inhibitory effect on CTGF expression (Figure 2B).

Further fluorescence analysis using FAM-labeled ASOs showed that the fluorescence of unmodified ASO sequences almost disappeared by day 5 post-transfection, whereas the fluorescence of modified CTGF-ASOs persisted through day 7 (Figure 2C). This suggests that modified CTGF-ASOs remain in the cells longer, likely due to increased resistance to ribonuclease degradation caused by the modifications.

To explore the impact of modified ASOs on PSF cell proliferation, we conducted EdU assays. The results (Figure 2D) confirmed our expectations: both MOE and LNA-modified CTGF-ASOs significantly inhibited PSF cell proliferation. Notably, the LNA-modified ASO required a significantly lower concentration than the MOE-modified ASO to achieve the same level of cell proliferation inhibition. To exclude the cytotoxicity caused by high concentrations, we performed the CCK-8 assay to evaluate the cytotoxicity of ASO#1, MOE-ASO#1, LNA-ASO#1 and scrambled ASO at concentrations of 10, 50, 100, 250, 500, and 1,000 nM, as shown in Supplementary Figures S3A-D. The cell viability of ASO#1 or scrambled control at the tested concentrations did not show significant reduction, indicating that no cytotoxicity was observed under these conditions.

Collagen, the most abundant protein in the skin, is crucial for maintaining skin structure and function (Epstein and Munderloh, 1978; Huang et al., 2024). We focused on the mRNA expression levels of type I and III collagen. The results (Figures 2E–I,L) showed that MOE and LNA-modified CTGF-ASOs dose-dependently inhibited the expression of Col-1α, Col-3α mRNA and protein. While also inhibited the expression of α-SMA, and fibronectin (Fn) mRNA and protein in PSF Cells. Additionally, LNA-modified ASOs demonstrated superior inhibitory effects at the same molar concentrations compared to MOE-modified ASOs (Figures 2E–H).

We also examined the effects of MOE and LNA-modified CTGF-ASOs on the TGF-β1/Smad and Non-Smad signaling pathways, which regulate fibrosis. TGF-β1 is a key regulator of fibrosis, influencing its progression through these pathways (Hao et al., 2023). The results (Figures 2J,K,M,N) indicated that MOE and LNA-modified CTGF-ASOs significantly inhibited the phosphorylation levels of Smad 2/3, PI3K, AKT, and ERK in the cells.

In summary, the MOE and LNA modifications enhanced the stability and efficacy of CTGF-ASOs, effectively inhibiting PSF cell proliferation, ECM protein expression, and key fibrosis-related signaling pathways. These findings highlight the therapeutic potential of modified CTGF-ASOs in scar treatment.

### 3.3 MOE/LNA-ASO#1 reduces scar formation in rabbit ear model

**FIGURE 3:**
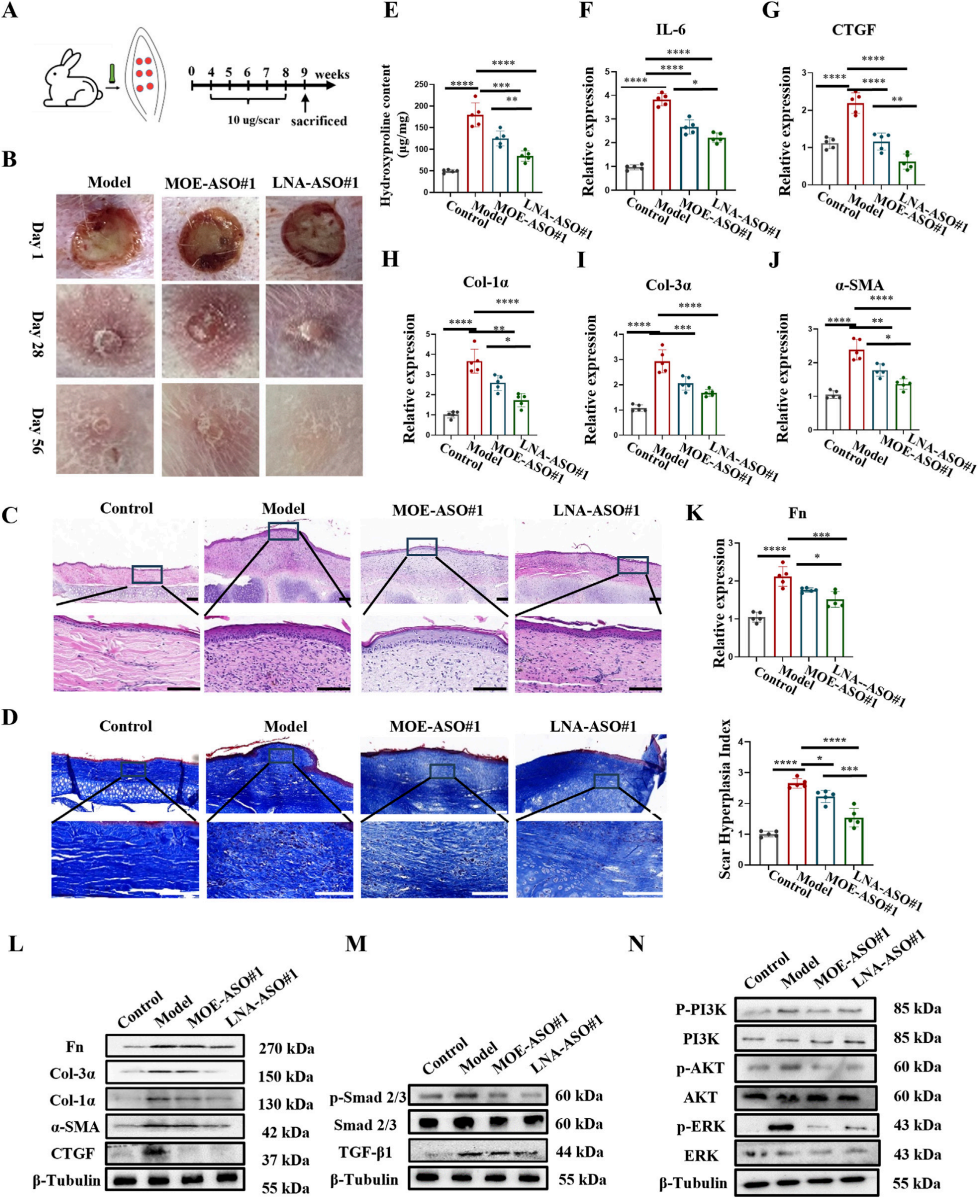
MOE/LNA-ASO#1 Reduces Scar Formation in Rabbit Ear Model **(A)** Schematic diagram of rabbit ear scar model. **(B)** The appearance of rabbit ear scar changes in different periods. **(C)** HE staining results. **(D)** Masson staining results. **(E)** Hydroxyproline content. **(F)** Effect of MOE\LNA-ASO#1 on IL-6 expression level. In rabbit ear tissue, the effect of MOE\LNA-ASO#1 on the mRNA expression and protein expression of CTGF **(G)**, Col-1α **(H)**, Col-3α **(I)**, α -SMA **(J)** Fn **(K)** mRNA and protein **(L)** Effect of MOE\LNA-ASO#1 on Smad 2/3 **(M)** PI3K, AKT and ERK phosphorylation level **(N)** signaling pathway proteins. Scale bars represent 50 μm*p < 0.05, **p < 0.01, ***p < 0.001, ****p < 0.0001.

To evaluate the biological function of ASOs *in vivo*, we established a rabbit ear scar model (Figure 3A); (Ren et al., 2013). By day 28 post-surgery, prominent hypertrophic scars developed on the rabbit ears, characterized by raised, firm, and reddish scar tissue in the model group (Figure 3B; Supplementary Figure S4A). HE staining (Figure 3C; Supplementary Figure S4B) revealed thickened epidermis, numerous new capillaries, inflammatory cells, and fibroblasts in the dermis of the scar tissue. Masson staining (Figure 3D) showed disorganized, nodular, or whorled collagen fibers.

After 4 weeks of treatment with MOE-ASO#1 or LNA-ASO#1, the height of the scar protrusions decreased, and the color lightened. HE staining showed reduced dermal thickness and fewer inflammatory cells and fibroblasts post-treatment. Masson staining indicated that collagen fibers, which appeared dense and disordered in the model group, became more organized following treatment. Additionally, hydroxyproline content was lower in the treated groups compared to the model group (Figure 3E). To assess the anti-inflammatory effect of CTGF-ASO in scars, we measured IL-6 levels in the scar tissue, finding that CTGF-ASO treatment reduced IL-6 production (Figure 3F). In the comparison of the efficacy between MOE-ASO#1 and LNA-ASO#1, LNA-ASO#1 is more effective in treating scar proliferation.

qPCR and Western blot analysis revealed that both MOE and LNA-ASO#1 treatments significantly downregulated the expression of CTGF, type I collagen, type III collagen, α-SMA and Fn proteins in the scar tissue (Figures 3G–L) and LNA-ASO#1 is more effective.

To gain a more comprehensive understanding of the mechanism, we further examined the expression levels of proteins in the TGF-β1/Smad and Non-Smad signaling pathways. The phosphorylation levels of Smad 2/3, PI3K, AKT, and ERK were significantly inhibited by ASO treatment, consistent with the trends observed in ECM protein levels (Figures 3M,N).

### 3.4 MOE/LNA-ASO#1 effectively inhibits scar proliferation in a mouse model

**FIGURE 4:**
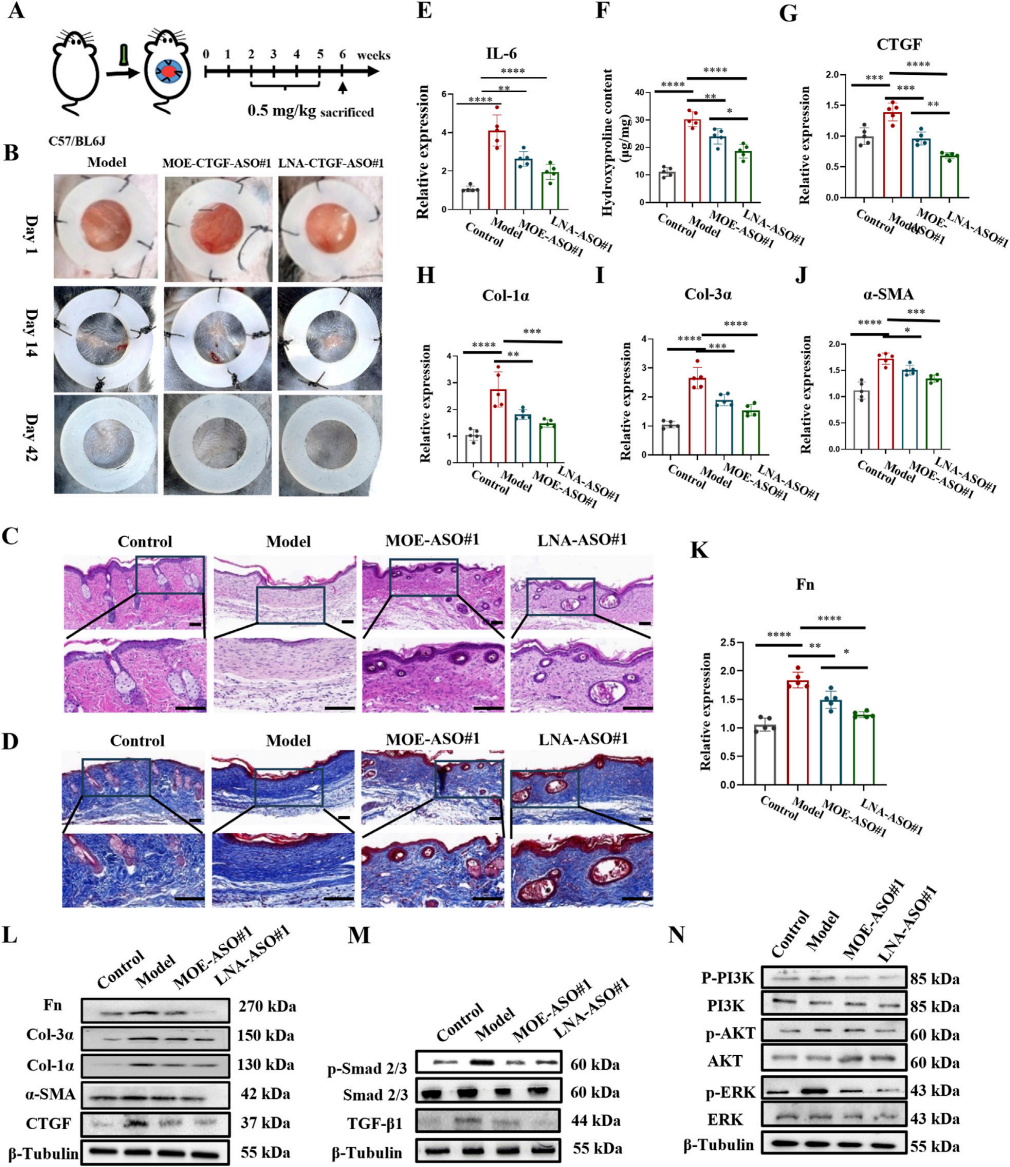
MOE/LNA-ASO#1 Effectively Inhibits Scar Proliferation in a Mouse Model **(A)** Schematic diagram of mouse hypertrophic scar model. **(B)** Changes in the appearance of scars in mice in different periods. **(C)** HE staining. **(D)** Masson staining. **(E)** Effect of MOE\LNA-ASO#1 on IL-6 expression level. **(F)** Hydroxyproline content. In mice, the effect of MOE\LNA-ASO#1 on the mRNA expression of CTGF **(G)** Col-1α **(H)** Col-3α **(I)** α -SMA **(J)** FN **(K)** mRNA and protein **(L)** Effect of MOE\LNA-ASO#1 on Smad 2/3 **(M)** PI3K, AKT and ERK phosphorylation level **(N)** signaling pathway proteins. Scale bars represent 50 μm *p < 0.05, **p < 0.01, ***p < 0.001, ****p < 0.0001.

In addition, we established a mouse hypertrophic scar model as shown in Figure 4A (Li et al., 2019). Scar formation was induced by applying surface tension to a silicone ring sutured onto the wound. On day 14 post-surgery, wounds were healed, and intralesional treatments began. Data were collected on day 42 post-surgery. As seen in Figure 4B and Supplementary Figure S5A, the model group displayed large scar areas, whereas the treatment groups exhibited significantly reduced scar areas and new hair growth.

Histological analysis using HE, Masson and Sirius Red Staining was performed on the scar tissue sections to observe morphological changes and collagen structure. As shown in Figures 4C,D, Supplementary Figure S5B, the model group scars lacked hair follicles, sebaceous glands, and other dermal appendages. After 4 weeks of treatment with MOE-ASO#1 or LNA-ASO#1, the scar areas were reduced, and skin morphology resembled normal tissue, with the reappearance of hair follicles and sebaceous glands. Hydroxyproline content was lower in the treated groups compared to the model group (Figure 4F). To assess the anti-inflammatory effects of CTGF-ASO in scars, IL-6 levels were measured in the scar tissue, and CTGF-ASO treatment reduced IL-6 production (Figure 4E).

Further analysis showed significant downregulation of CTGF, type I collagen, type III collagen, and α-SMA mRNA and protein expression in mouse scar tissue following ASO treatment (Figures 4G–L). In the comparison of the efficacy between MOE-ASO#1 and LNA-ASO#1, LNA-ASO#1 is more effective in treating scar proliferation (Figures 4G–K).

Mechanistic studies revealed significant changes in the TGF-β1/Smad and Non-Smad signaling pathways. ASO treatment inhibited TGF-β1-induced Smad signaling, as indicated by reduced phosphorylation levels of Smad 2/3. Additionally, the PI3K, AKT, and ERK signaling pathways were effectively inhibited (Figures 4M,N). These findings confirm the potent antifibrotic activity of MOE and LNA-ASO#1.

### 3.5 MOE/LNA-ASO#1 effectively inhibits human keloid growth in nude mice

**FIGURE 5:**
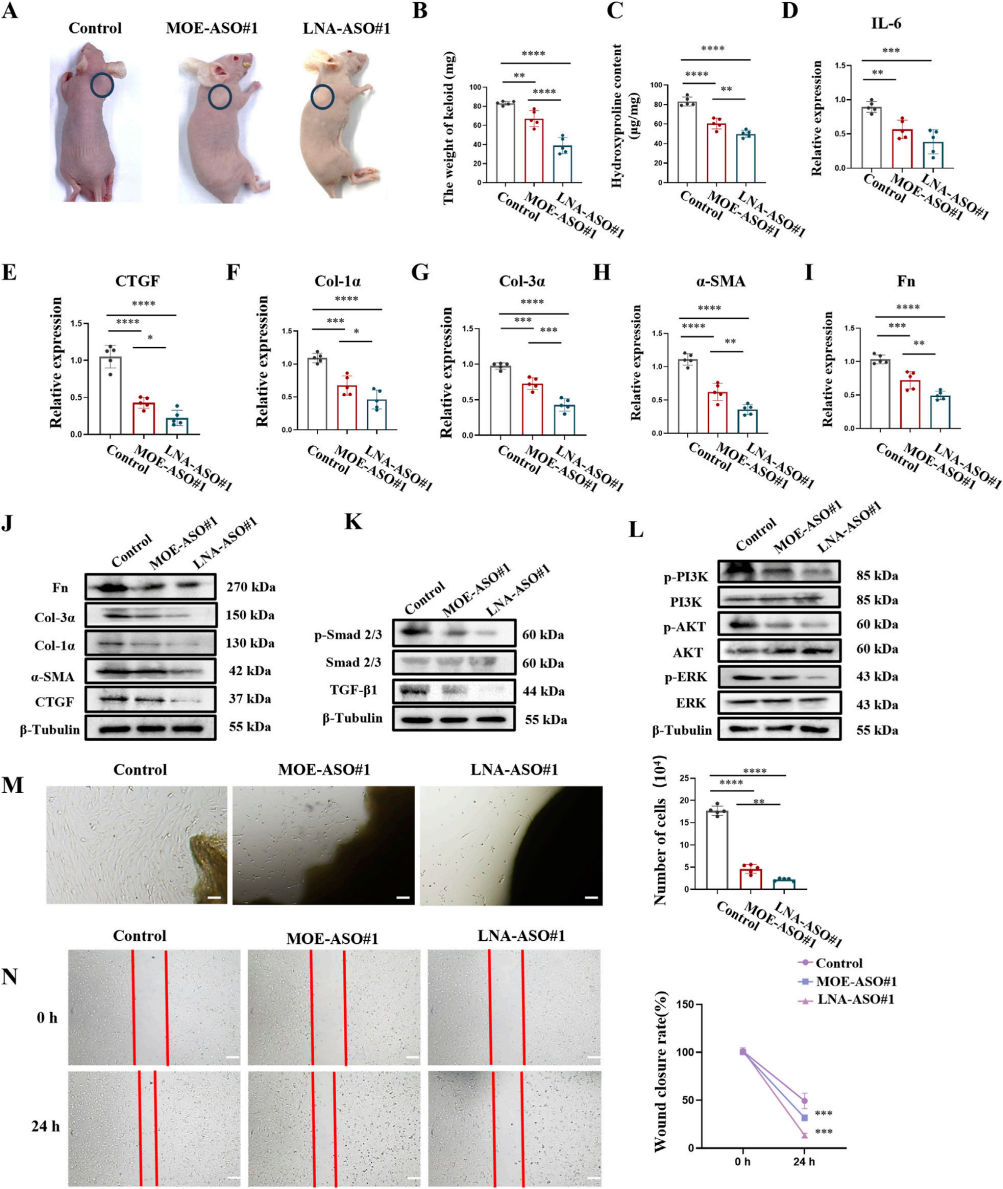
MOE/LNA-ASO#1 Effectively Inhibits Human Keloid Growth in Nude Mice **(A)** Appearance of Keloid after medication administration. **(B)** Keloid weight. **(C)** Hydroxyproline content. **(D)** Effect of MOE\LNA-ASO#1 on IL-6 expression level. After ASO treatment, the expressions of CTGF **(E)** COL-1 α **(F)** COL-3 α **(G)** α -SMA **(H)** FN **(I)** mRNA and protein **(J)** were detected. Smad 2/3 **(K)** PI3K, and ERK phosphorylation levels **(L)** signaling pathway proteins. KF cell proliferation **(M)** and scratch assay **(N)** Scale bars represent 100 μm *p < 0.05, **p < 0.01, ***p < 0.001, ****p < 0.0001.

To more accurately simulate the effects of MOE/LNA-ASO#1 in humans, we established a nude mouse keloid xenograft model (Li et al., 2019). This model preserves the complex interactions between human cells in both the epidermis and dermis, making it a close approximation of *in vivo* human tissue studies. The results showed that treatment with MOE/LNA-ASO#1 significantly reduced the volume and weight of the keloid tissues compared to untreated controls (Figures 5A,B; Supplementary Figure S6). The weight of keloids treated with LNA-ASO#1 was lower than those treated with MOE-ASO#1. Additionally, hydroxyproline content was lower in the treated groups than in the model group (Figure 5C), indicating reduced collagen content. To assess the anti-inflammatory effects of CTGF-ASO in scars, IL-6 levels were measured in the keloid and MOE/LNA-ASO#1 treatment reduced IL-6 production (Figure 5D). And, LNA-ASO#1 was more effective than MOE-ASO#1 in treating keloids.

Further gene expression analysis of the keloid tissues revealed that ASO treatment dose-dependently reduced the expression of CTGF, α-SMA, Col-1α, Col-3α, and Fn (Figures 5E–J). This indicates that ASOs inhibited the production of ECM proteins in keloid tissues. Additionally, the phosphorylation levels of Smad 2/3, PI3K, AKT, and ERK were significantly reduced following ASO treatment, demonstrating effective inhibition of these signaling pathways (Figures 5K,L). These results suggest that ASOs downregulate the expression of fibrosis-related pathogenic genes in skin fibroblasts by inhibiting the TGF-β1/Smad and non-Smad signaling pathways.

To further validate the inhibitory effects of ASOs on keloid fibroblast (KF) proliferation and migration, we established an *ex-vivo* keloid culture model. In the untreated group, KF cells migrated out from the edge of the keloid explants and spread across the culture dish within a week. In contrast, MOE/LNA-ASO#1 treatment effectively inhibited KF cell proliferation and migration (Figures 5M,N).

## 4 Discussion

The management of pathological scars, including hypertrophic scars and keloids, remains a significant challenge in clinical practice due to their complex pathophysiology and the limitations of existing treatments (Marneros and Krieg, 2004; Oettgen and Haubner, 2022). Current therapeutic strategies, ranging from surgical interventions to pharmacological treatments, often yield inconsistent outcomes and can be associated with substantial side effects (Wolfram et al., 2009; Gauglitz et al., 2011; Ekstein et al., 2021). This study provides compelling evidence for the efficacy of ASOs targeting CTGF as a novel and promising approach for scar management.

CTGF is a critical mediator in the fibrotic pathway, acting downstream of TGF-β1 to promote fibroblast proliferation and ECM production, which are key processes in scar formation (Castleberry et al., 2016; Jun et al., 2021). By specifically targeting CTGF, our study addresses a pivotal component of the fibrotic cascade, potentially offering more precise and effective intervention compared to broader TGF-β1 inhibitors. The use of ASOs enables targeted downregulation of CTGF expression, providing a mechanism to modulate pathological processes without completely inhibiting essential wound healing functions of TGF-β1 (Barrientos et al., 2008; Ramazani et al., 2018; Yang et al., 2022).

Among the 11 ASOs targeting exon five of CTGF, ASO#1 demonstrated a concentration-dependent inhibition of CTGF expression and significantly suppressed fibroblast activation and extracellular matrix production (including reductions in Col-1α, Col-3α, and α-SMA levels). Due to its potent antifibrotic effects, ASO-1 was selected as the lead candidate for further investigation. The introduction of MOE and LNA modifications significantly improves the stability and efficacy of CTGF-ASOs (Masaki et al., 2018; Bockstahler et al., 2022; Jin and Zhong, 2023). MOE modification, by introducing a 2′-O-methyl-oxyethyl group at the ribose 2′position, enhances the resistance of ASOs to nucleolytic degradation and improves their overall stability *in vivo* (Sheng et al., 2020). This modification also strengthens the binding affinity of ASOs to their target mRNA, prolonging their functional duration. On the other hand, LNA modification involves a conformational change of the sugar ring in the nucleic acid backbone, which locks the 2′-oxygen atom and confers increased rigidity to the structure (Swayze et al., 2007; Kuespert et al., 2020). This modification leads to a significant enhancement in the binding specificity and affinity of ASOs to their target mRNA, thereby improving their therapeutic potential.

Our data indicate that LNA-ASO#1 exhibits superior inhibitory effects on fibroblast proliferation, ECM production, and key signaling pathways involved in fibrosis, compared to MOE-ASO#1. This suggests that while both MOE and LNA modifications enhance ASO stability and efficacy, LNA modification provides an additional advantage by amplifying the binding specificity and affinity to the target mRNA, resulting in a stronger therapeutic effect. Consequently, LNA-ASO#1 emerges as a more potent candidate, with enhanced therapeutic effectiveness in fibrosis treatment.

Histological analyses revealed that treated scar tissues exhibited more organized collagen fibers, reduced fibroblast activity, and lower inflammatory cell infiltration, indicative of effective scar remodeling. The inhibition of Smad 2/3, PI3K, AKT, and ERK phosphorylation by CTGF-ASOs underscores their role in modulating both Smad-dependent and independent fibrotic signaling pathways, providing a comprehensive antifibrotic effect.

The promising results of this study pave the way for further investigations into the broader therapeutic applications of CTGF-ASOs. Given the role of CTGF in various fibrotic diseases, such as pulmonary fibrosis, cardiac fibrosis, and systemic sclerosis, there is significant potential for these ASOs to be adapted for treating other fibrotic conditions (Toda et al., 2018; Fu et al., 2022; Qiu et al., 2024). Ongoing research in our laboratory is exploring these possibilities, aiming to extend the benefits of CTGF-ASO therapy beyond dermal scarring.

Moreover, the integration of CTGF-ASOs into existing scar management protocols could revolutionize current treatment paradigms. Combining CTGF-ASOs with other therapeutic modalities, such as laser therapy, silicone gel sheeting, or corticosteroid injections, may enhance overall treatment efficacy and patient outcomes.

However, it is important to note that our study is only a preliminary step, and there remains substantial work to be done before these therapies can be translated into broader clinical applications. Key areas that need further investigation include the evaluation of potential toxicity and a comprehensive pharmacokinetic (PK) profile of CTGF-ASOs. These assessments are critical for determining the long-term safety and efficacy of this therapeutic strategy, especially for its potential use in diverse fibrotic diseases. Currently, ongoing studies in our lab are focused on evaluating these critical parameters, which will provide valuable insights into the optimal dosing, administration routes, and potential side effects associated with CTGF-ASO therapy.

In conclusion, this study demonstrates that LNA-modified CTGF-targeting ASOs represent a highly effective and safe therapeutic strategy for reducing scar formation. By specifically inhibiting a key mediator in the fibrotic pathway, these ASOs offer a targeted approach with the potential to significantly improve the quality of life for patients suffering from pathological scarring. However, further clinical development and trials are warranted to fully realize the therapeutic potential of CTGF-ASOs in scar management and other fibrotic diseases.

## Significance statement

This study designed 11 antisense oligonucleotides (ASOs) targeting the fifth exon of CTGF, followed by modifications and screening. Notably, CTGF-ASO#1 demonstrated significant inhibition of fibroblast proliferation and extracellular matrix protein expression both *in vitro* and *in vivo*, effectively reducing scar formation. These findings hold promise for the development of a novel therapeutic agent aimed at targeting the CTGF signaling pathways to mitigate scar formation, offering potential clinical applications.

## Data Availability

The original contributions presented in the study are included in the article/Supplementary Material, further inquiries can be directed to the corresponding authors.
