# Supplementary material for: Locked nucleic acid-modified antisense oligonucleotides attenuate scar hyperplasia through targeted inhibition of CTGF
Source: Front Pharmacol. 2025 Aug 21;16:1623640. doi: 10.3389/fphar.2025.1623640 (PMC12408595; doi:10.3389/fphar.2025.1623640)
Supplement: Supplementary file 1 [file Presentation1.pdf]

# Supplementary materials

**Locked Nucleic Acid-Modified Antisense Oligonucleotides Attenuate Scar Hyperplasia through Targeted Inhibition of CTGF**

Jinhe Li[1], Xi Wu[1], Ying Yang[1], Ruiqi Mao[1], Zherui Li[1], Xiujun Zhang[3], Wenguo Wei[4], Wendi Wang[2*], Hailong Li[1*], Honggang Zhou[1*], Cheng Yang[1,5 *]

[1] College of Pharmacy, State Key Laboratory of Medicinal Chemical Biology, Nankai University, Tianjin 300350, China;[2] Department of Plastic and Burn Surgery, Tianjin First Central Hospital, No.24 Kangfu Road, Nankai District, Tianjin, 300192, China;[3] Department of Dermatology, Tianjin Academy of Traditional Chinese Medicine Affiliated Hospital, Tianjin 300120, China;[4] Department of Dermatology, Tianjin First Central Hospital, School of Medicine, Nankai University, Tianjin 300192, China; [5]Nankai International Advanced Research Institute(Shenzhen Futian), ShenZhen 518045, China

**Runing title:** LNA-modified CTGF-ASO inhibit scar hyperplasia

* Corresponding Author

Address for correspondence: Honggang Zhou; College of Pharmacy, State Key Laboratory of Medicinal Chemical Biology, Nankai University, Tianjin 300350, China; Phone: 022-85358566; honggang.zhou@nankai.edu.cn.

**Supplemental Figures**

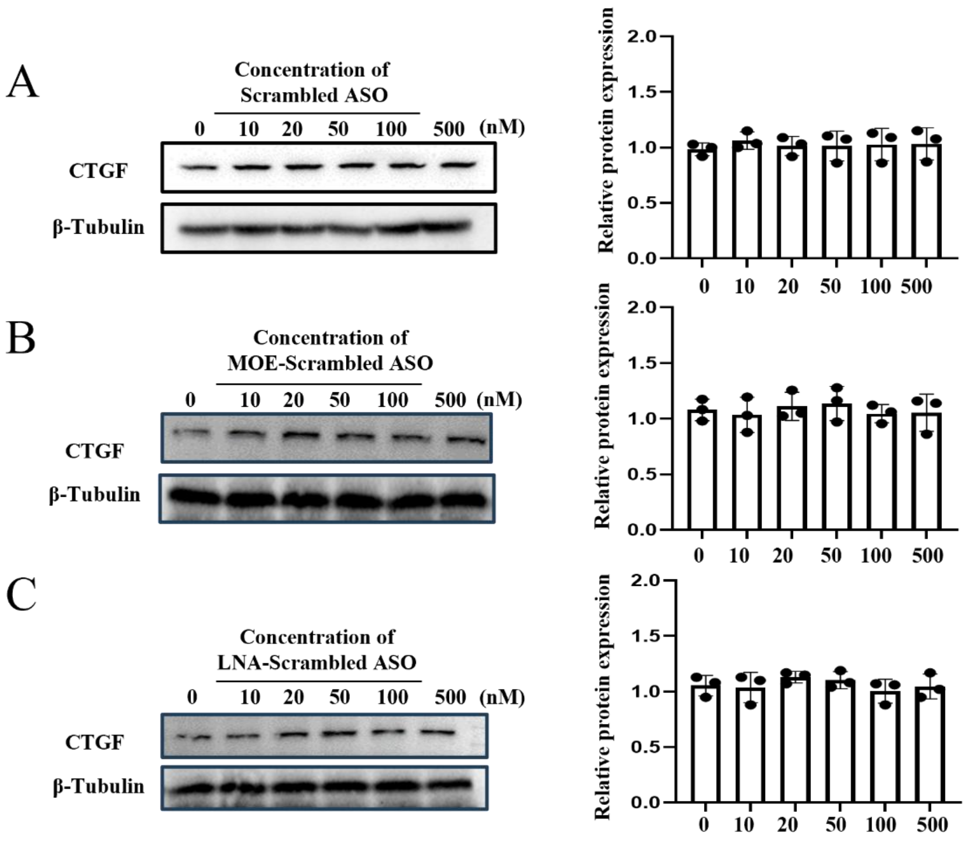


**Supplemental Fig. 1:** Effects of Scrambled ASO, MOE-Scrambled ASO and LNA-Scrambled ASO on CTGF Protein Expression. Effect of Scrambled ASO (A), MOE-Scrambled ASO (B), and LNA-Scrambled ASO (C) on CTGF Protein Expression Under Varying Concentration Gradients.

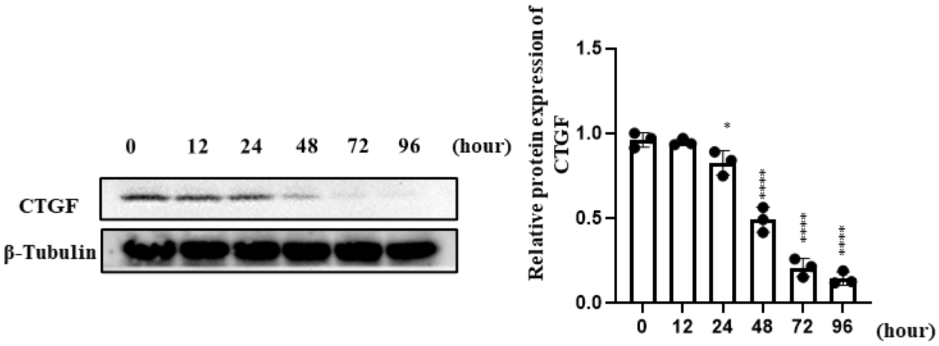


**Supplemental Fig. 2：** Time dependent inhibition of CTGF-ASO#1 on CTGF protein expression at 50 nM. *p < 0.05, **p < 0.01, ****p < 0.0001.

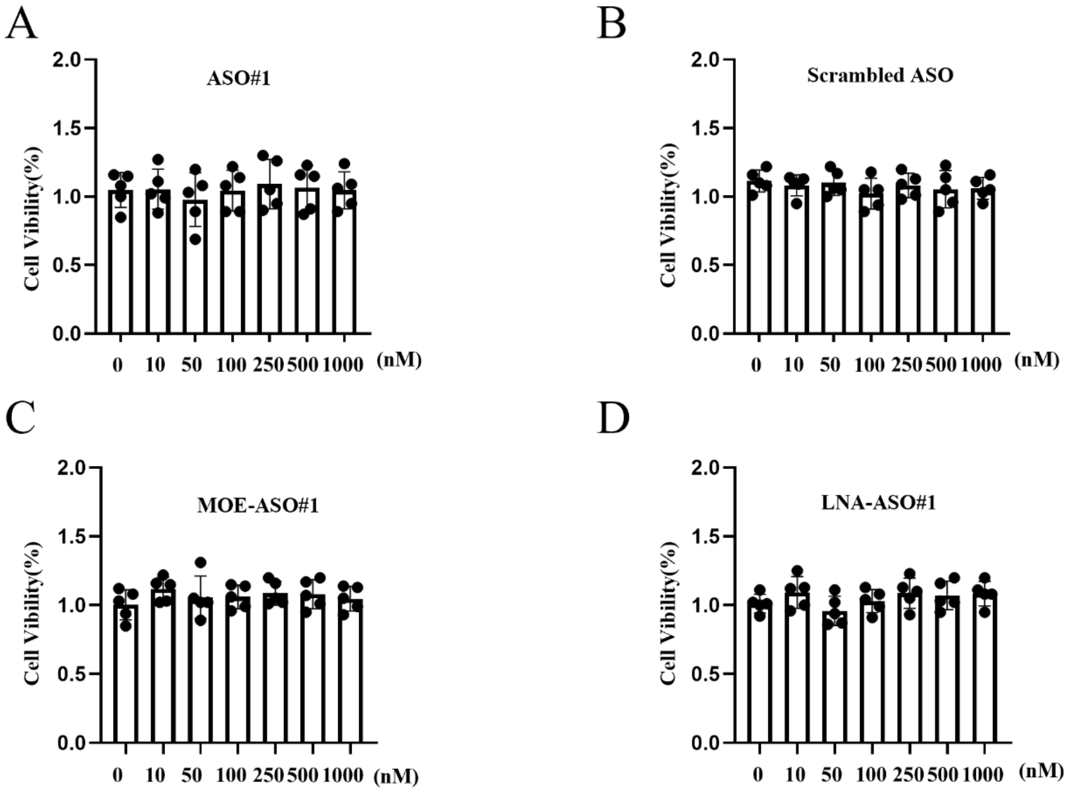


**Supplemental Fig. 3：** Evaluation of Cytotoxic Effects of ASO Treatments by CCK8 Assay (A)ASO#1. (B) Scrambled ASO. (C)MOE-ASO#1. (D)LNA-ASO#1.

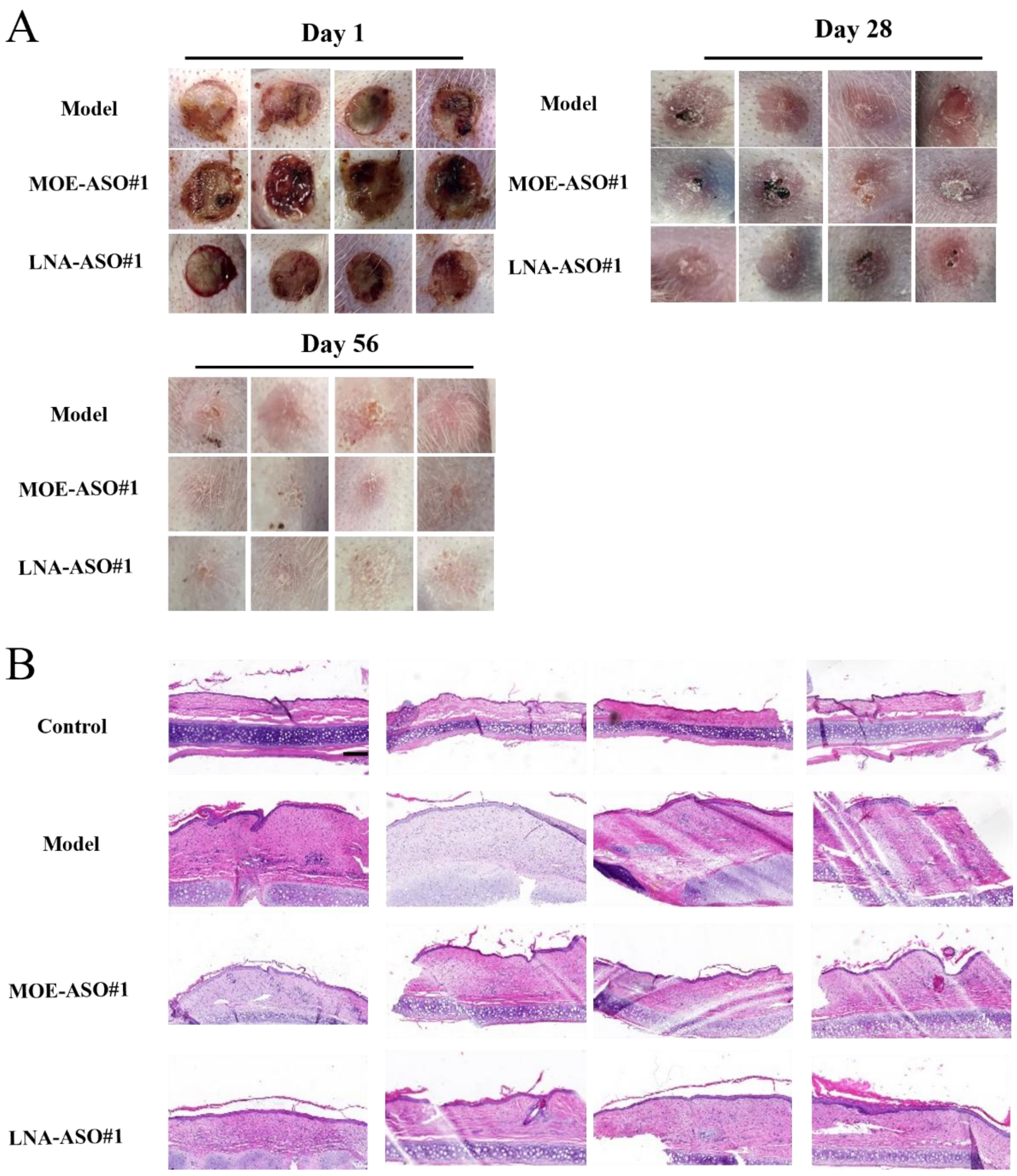


**Supplemental Fig. 4:** Effect of MOE\LNA-ASO#1 on hypertrophic scar in Rabbit Ear Model

(A) Appearance of rabbit ear scars at different times (1,28 and 56 days). (B) HE staining results. Scale bars represent 50 μm.

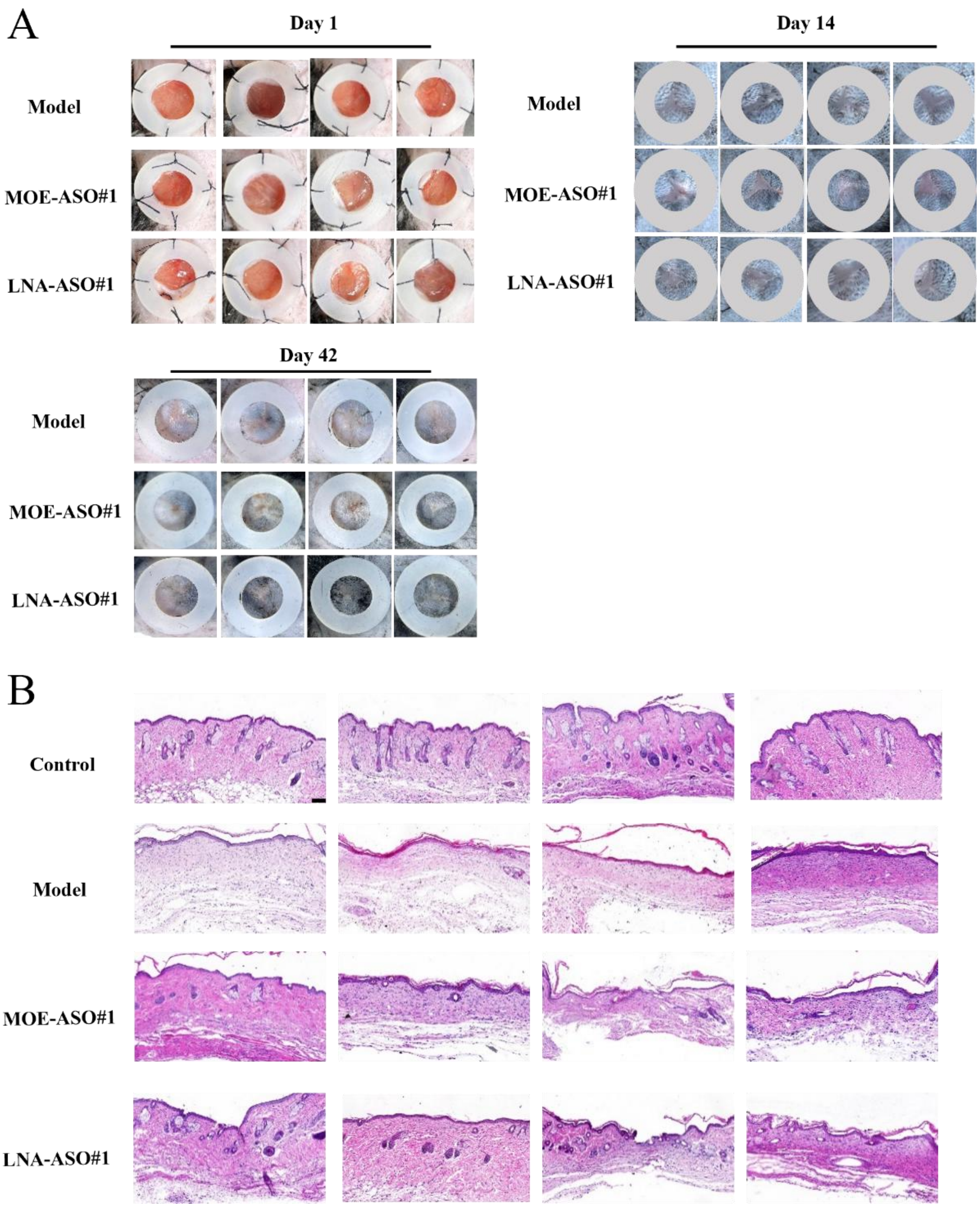


**Supplemental Fig. 5:** Effect of MOE\LNA-ASO#1 on hypertrophic scar in mice

(A) Appearance of mice ear scars at different times (1,14 and 42 days). (B) HE staining results. Scale bars represent 50 μm.

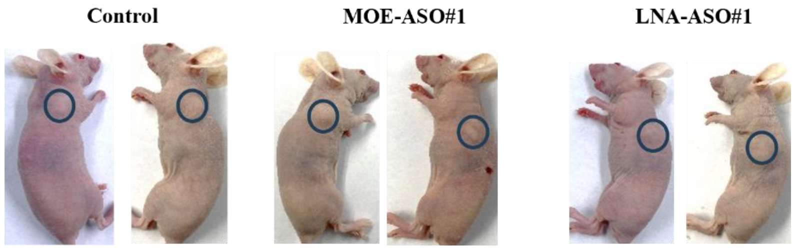

**Supplemental Fig. 6：** Appearance of Keloid after medication administration
